# Toxic effects of dimethyl sulfoxide on red blood cells, platelets, and vascular endothelial cells *in vitro*

**DOI:** 10.1002/2211-5463.12193

**Published:** 2017-02-20

**Authors:** Xiaoyang Yi, Minxia Liu, Qun Luo, Hailong Zhuo, Hui Cao, Jiexi Wang, Ying Han

**Affiliations:** ^1^ Beijing Institute of Transfusion MedicineBeijingChina; ^2^ Department of TransfusionAffiliated Hospital of Academy of Military Medical SciencesBeijingChina; ^3^ Beijing Red Cross Blood CenterBeijingChina

**Keywords:** dimethyl sulfoxide, EAhy926 cells, platelets, red blood cells

## Abstract

Dimethyl sulfoxide (DMSO) is widely used in biological studies as a cryoprotective agent for cells and tissues, and also for cryopreserved platelets (PLTs). However, few data on the toxic effects of DMSO following intravenous infusion of cryopreserved PLTs are available. The aim of this study was to explore dose‐related effects of DMSO on red blood cells (RBCs), PLTs and vascular endothelial cells *in vitro*. The results showed that DMSO treatments had significant effects on RBCs, affecting osmotic fragility and increasing hemolysis. Free hemoglobin (FHb) level of RBCs was 0.64 ± 0.19 g L^−1^ after incubation for 6 h with 0.6% DMSO, and these levels were elevated compared with controls (0.09 ± 0.05 g L^−1^). Aggregation of PLTs induced by adenosine diphosphate, thrombin (THR), and thrombin receptor activator peptide (TRAP) were inhibited by DMSO treatment because the THR generation capacity was reduced. The intensity of the cytosolic esterase‐induced fluorescence response from carboxy dimethyl fluorescein diacetate (CMFDA) in PLTs was decreased about 29% ± 0.04% after treatment with DMSO. DMSO also inhibited the proliferation of the vascular endothelial cell line EAhy926 cells by blocking the G1 phase. Apoptosis of EAhy926 cells with 0.6% DMSO stimulation was increased threefold compared to controls. On the basis of these findings, it was concluded that DMSO was toxic to the hematologic system. This should be taken into account when assessing the infusion effects of cryopreserved PLTs or other blood products requiring DMSO as a vehicle, such as cryopreserved stem cells, in order to avoid adverse therapeutic effects.

**Abbreviations**
ACD: anticoagulant citrate dextrose
CMFDA: carboxy dimethyl fluorescein diacetate
DMSO: dimethyl sulfoxide
FHb: free hemoglobin
PI: propidium iodide
PLTs: platelets
PRP: platelet‐rich plasma
RBCs: red blood cells
TF: recombinant human coagulation factor III/tissue factor
THR: thrombin
TRAP: thrombin receptor activator peptide

Dimethyl sulfoxide [DMSO, (CH_3_)_2_SO], a highly polar organic liquid that has one polar sulfinyl domain and two apolar methyl groups, is used widely as a chemical solvent 1, 2. Because of its ability to penetrate biological membranes, it is used as an agent for hypothermal storage of blood cells, including the cryopreservation of platelets (PLTs) 3, 4. DMSO can readily penetrate into living cells and modulates membrane permeability, which gives it cryoprotective property 5. DMSO decreases the formation of intracellular ice crystals; therefore, frozen injuries of cells and tissues mixed with DMSO can be reduced 6. In 1962, Iossifides and Tocantins found short‐lived preservation of clot retraction activity of PLTs frozen with DMSO *in vitro*, and since then DMSO has been the classic cryoprotective vehicle for PLTs 7.

In general, DMSO has low acute and chronic toxicity for animal, plant, and aquatic life 8, 9. According to the reports, *in vivo* studies investigating the metabolic fate of DMSO in human indicate that DMSO is rapidly absorbed, reaching a peak in serum at 4–8 h after oral or transcutaneous administrations, is cleared from the blood within 120 h after ingestion of a single dose, and is primarily excreted in urine unchanged or as DMSO_2_ (the major metabolite found in human) 10, 11, 12, 13.

The United States Food and Drug Administration (FDA) has not approved cryopreserved PLTs for clinical use when DMSO is used as the cryoprotective agent. Although clinic studies have reported that PLT products cryopreserved with DMSO were infused into the human body, the effects have been controversial. It has been reported that cryopreserved PLTs can be used in emergency circumstances, but infusion of cryopreserved PLTs is less effective than fresh PLTs 14. Long‐term exposure to respiratory irritants may result in disease of the airways involving difficulty breathing and related systemic problems 8, 9. Massive intravenous infusion with DMSO cryopreserved products can cause occasional skin irritation, garlicky breath and body odor, and liver and kidney dysfunction. There have been reports of leukemia patients presenting with abdominal pain, nausea, headache, bradycardia, and other symptoms, even death 15, 16, 17, 18, 19, 20, 21, 22. These investigations were mostly restricted to the skin, oral cavity, and respiratory tract, less is known about effects on the hematologic system.

In our studies of the infusion effects of cryopreserved PLTs, DMSO is employed to protect PLTs. Thus, it was important for us to evaluate adverse effects of DMSO itself on the hematologic system. For that purpose, we investigated the effects of DMSO on the membrane stability and function of blood cells and the proliferation of vascular endothelial cells involved in the infusion response, using *in vitro* blood cells and EAhy926 cells stimulated with DMSO or without DMSO (control).

## Materials and methods

### Main antibodies and reagents

Cell proliferation assay (MTS) solution was obtained from PreGene (Beijing, China). A plasma‐free hemoglobin (FHb) detection kit was obtained from Real‐Tech (Beijing, China). CellTracerTM Green CMFDA (5‐chloromethylfluorescein diacetate) was obtained from Life Technologies (Eugene, Oregon, USA). DMSO, adenosine diphosphate (ADP), thrombin (THR), and thrombin receptor activator peptide (TRAP) were obtained from Sigma (St. Louis, MO, USA). Annexin V‐FITC Kit and cell cycle detection kit was obtained from KeyGEN BioTECH (Nanjing, China). Corn Trypsin Inhibitor was obtained from Absin Bioscience (Shanghai, China). Anti‐Human CD41a FITC was obtained from eBioscience (San Diego, CA, USA). Recombinant human coagulation factor III/tissue factor (TF) was obtained from Bio‐techne (MN, USA). THR substrate S2238 was obtained from Eurogentec (Liege, Belgium).

### Cell sample preparation

The study was approved by the Ethics Committee of the Academy of Military Medical Sciences and all aspects of the study comply with the Declaration of Helsinki. Ethics Committee of the Academy of Military Medical Sciences specifically approved that no informed consent was required because data were going to be analyzed anonymously. The whole blood, red blood cells (RBCs), and platelet‐rich plasma (PRP) samples from healthy adults with anticoagulant citrate dextrose solution (ACD) as the anticoagulant were from the Beijing Red Cross Blood Center. PLTs were suspended in autologous plasma to a final cell density of 3 × 10^8^ mL^−1^ and allowed to rest 60 min at room temperature before experiments. RBCs had no other treatment. Human umbilical vascular endothelial cells line EAhy926 cells were derived from Beijing Institute of Transfusion Medicine (China).

### DMSO concentration selection

According to the following reports 23, 24, 25, cryopreserved PLTs were infused into the human body without removing 6% DMSO centrifugally. The volume of 1 U cryopreserved PLTs was 250–300 mL, the blood volume of a 60‐kg patient (7% of body weight) is about 4200 mL, computational formula of DMSO final concentration is presented as following:

$$\begin{array}{cc} \%\;\text{DMSO}=\;\; & \frac{\text{Volume (1 U cryopreserved PLTs)}\times \text{DMSO concentration}}{\text{Whole blood volume (a 60 kg patient)}}\times 100\%\\ & =\frac{250\mathrm{mL}\times 6\%}{4200\mathrm{mL}}\times 100\%\approx 0.4\% \end{array}$$

Finally, we selected 0.2%, 0.4%, and 0.6% as the study concentration.

### Free hemoglobin and apoptosis of RBCs assay *in vitro*

Free hemoglobin levels were examined using the detection kit. Briefly, RBCs were gently mixed with or without DMSO (0.2%, 0.4%, and 0.6%), and then incubated at 37 °C for 0, 6, 12, 24, 48, and 72 h. FHb was measured by ultraviolet spectrophotometer (Persee, China). Cells were then stained with Annexin V‐FITC according to the manufacturer's protocol. Data were acquired using the Cytomics FC 500 flow cytometer (Beckman Coulter 175487, Fullerton, CA, USA) and analyzed on cxp software (Beckman Coulter).

### PLTs assay *in vitro*

Platelet aggregation in apheresis PLTs and whole blood was respectively measured by light transmission and impedance aggregometry (Model 700; Chronolog, Havertown, PA, USA) in response to 5 μm ADP, 1 U L^−1^ THR, and 10 μm TRAP. Samples were added into a reaction cup with continuous stirring at 37 °C, and then aggregation agonists were added to sample. After incubation for 6–9 min, PLT aggregation activity was measured, and the maximum aggregation rate was used to represent platelet aggregation activation. The corresponding platelet poor plasma (PPP) was used as the control in light transmission method.

For measurement of cytosolic esterase‐induced fluorescence intensity, PLTs were gently mixed with or without DMSO (0.2%, 0.4%, 0.6%), and then loaded with CMFDA at 10 μm and incubated for 30 min at 37 °C. Next, PLTs were washed with PBS and analyzed by laser confocal scanning microscope (Zeiss LSM 510, Oberkochen, Germany) and flow cytometry (Beckman Coulter 175487).

Before beginning a THR generation assay, whole blood samples were mixed with or without DMSO (0.2%, 0.4%, 0.6%), then lipidated tissue factor (110 pmol L^−1^) and CaCl_2_ (18 mmol L^−1^) were added to 0.35 mL of blood that had been prewarmed for 5 min at 37 °C. After mixing with the tissue factor and calcium, 10 μL subsamples of blood were transferred to individual tubes and kept at 37 °C. At specific times (every 0.5–4 min) for 40 min, 2 mL of ice‐cold buffer (0.1 mol L^−1^ NaCl, 0.04 mol L^−1^ Tris, 10 mol L^−1^ EDTA, 0.1% bovine serum albumin) was added to a tube. Each 10 μL blood sample was then compressed several times with a wooden stick, and the tube was placed on ice. After a 40‐min incubation period, diluted samples were removed by centrifugation. THR activity in the supernatants was measured by mixing 250 μL of each with 50 μL of 1 mmol L^−1^ S2238 in A405 after incubated at 37 °C for 15 min 26.

For measurement of CD41a content, PLTs were gently mixed with or without DMSO (0.2%, 0.4%, 0.6%) for 30 min, and then loaded with anti‐CD41a antibody at 5 μL and incubated for 15 min at 37 °C. Next, PLTs were washed with PBS and analyzed by flow cytometry (Beckman Coulter 175487).

Platelet apoptosis experiments were done using the same procedure described for RBCs.

### Vascular endothelial cells line EAhy926 cells assay *in vitro*

For the EAhy926 cell proliferation assay, EAhy926 cells were cultured in 96‐well plates at 4000 cells per well in RPMI‐1640 medium supplemented with 10% FBS. These cells were individually incubated with or without DMSO (0.2%, 0.4%, 0.6%). Cell appearance was observed daily by optical microscope. Cell proliferation was assayed by MTS assay for five consecutive days according to the manufacturer's protocol. The solubilized formazan dye product was quantified spectrophotometrically at 490 nm with a 96‐well plate reader.

For assay of EAhy926 cells cycle, EAhy926 cells were cultured in 96‐well plates at 4000 cells per well in RPMI‐1640 medium supplemented with 10% FBS. These cells were individually incubated with or without DMSO (0.2%, 0.4%, 0.6%). Cell cycle was assayed by cell cycle detection kit after 24 h of incubation according to the manufacturer's protocol. Data were acquired using the flow cytometer and analyzed on cxp software.

For assay of EAhy926 cells apoptosis, unfractionated EAhy926 cells were individually treated with or without DMSO (0.2%, 0.4%, 0.6%). Cells were then stained with Annexin V‐FITC and propidium iodide (PI) according to the manufacturer's protocol. Data were acquired using the flow cytometer and analyzed on cxp software.

### Statistical analysis

Experimental data were expressed as mean ± SEM. Each experiment was carried out at least three times. Statistical analyses for multiple‐group comparisons were performed by one‐way ANOVA, followed by *post hoc* Dunnett's test. A *P*‐value of < 0.05 was considered statistically significant.

## Results

### RBC hemolysis was increased after incubation with DMSO

**Figure 1:**
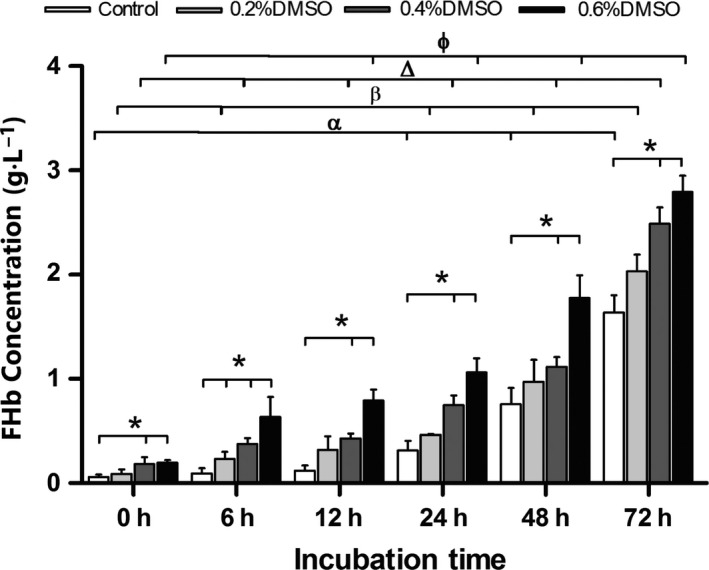
DMSO induces hemolysis in RBCs *in vitro*. DMSO concentrations were 0.2%, 0.4%, and 0.6%, and incubation time were 0, 6, 12, 24, 48, and 72 h. Controls were incubated without DMSO. Data are expressed as relative to control cells and are the mean ± SEM of three independent experiments. *P < 0.05, compared with control; ^α^ *P* < 0.05, control compared with 0 h; ^β^ *P* < 0.05, 0.2% DMSO compared with 0 h; ^Δ^ *P* < 0.05, 0.4% DMSO compared with 0 h; ^ϕ^ *P* < 0.05, 0.6% DMSO compared with 0 h.

Effects of DMSO on the hemolysis of RBCs *in vitro* were determined at different times (0, 6, 12, 24, 48, 72 h; Fig. 1). The basal level of FHb found in control cell supernatant was 0.06 ± 0.02 g L^−1^. In incubated RBCs, DMSO increased the supernatant FHb release in a dose‐dependent manner. Significant effects were observed with 0.2% DMSO at 6 h and with 0.4% DMSO at 0 h. Hemolysis levels were 0.23 ±0.07 g L^−1^, 0.37 ± 0.06 g L^−1^, and 0.64 ± 0.19 g L^−1^, respectively, for 0.2%, 0.4%, and 0.6% DMSO after incubation for 6 h, and these levels were elevated compared with control incubated for 6 h (0.09 ±0.05 g L^−1^). The longer the incubation with DMSO, the greater the content of FHb. Apoptosis levels of RBCs did not have any significant differences between control group and DMSO incubation groups (data not shown).

### PLT aggregation activity and cytosolic esterase‐induced fluorescence intensity were decreased after incubation with DMSO

**Figure 2:**
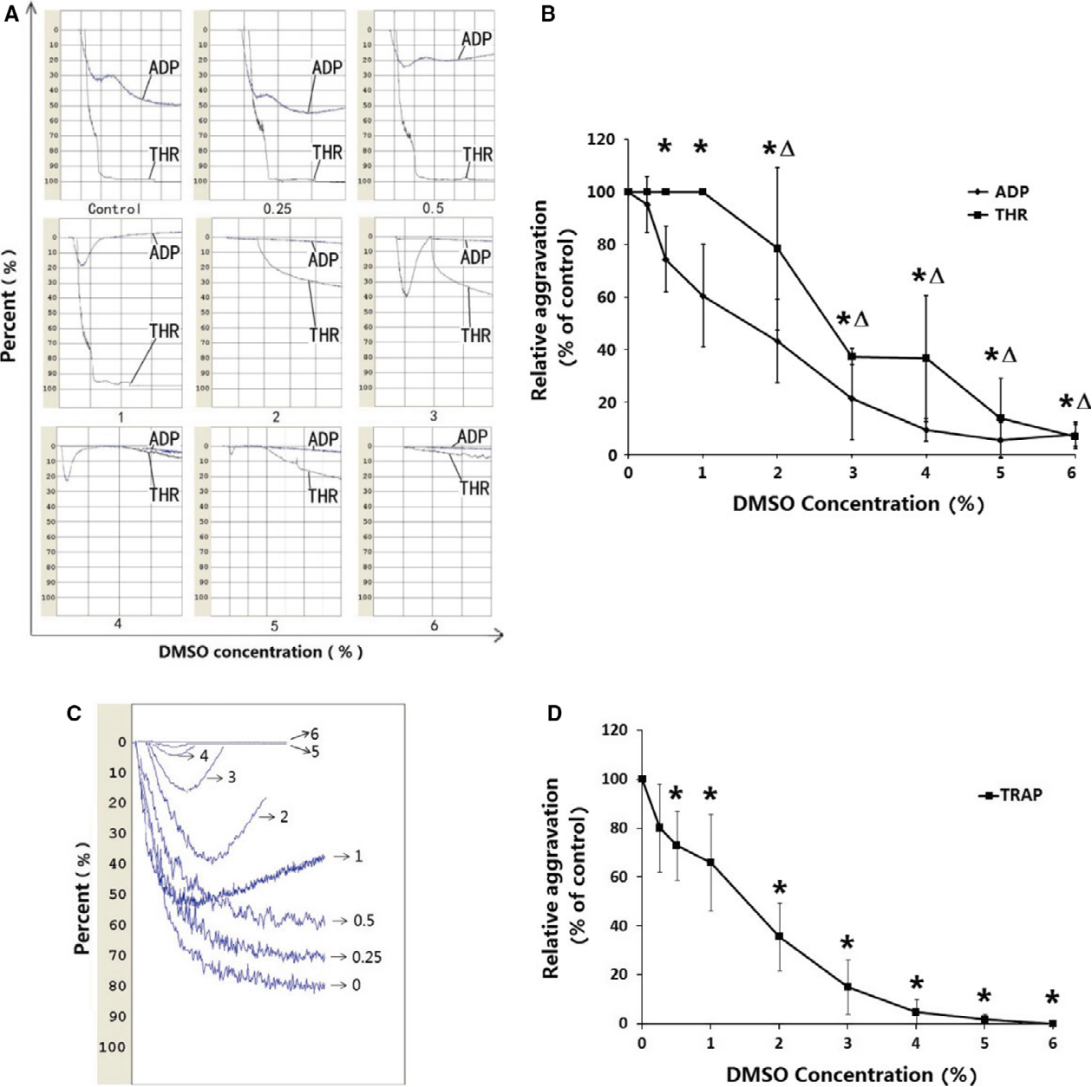
DMSO reduces aggregation rates of PLTs *in vitro*. DMSO concentrations were from 0.25% to 6%, and incubation time was 30 min. Controls were incubated without DMSO. Three aggregation stimuli ADP 10 U L^−1^, THR 1 U L^−1^ and TRAP 10 μm were used. (A, C) ADP,THR,TRAP were added to PLTs incubated with DMSO at different concentrations. Then samples were analyzed by light transmission aggregometry. (B, D) Quantitative data from three experiments are shown (mean ± SD). (B) *P < 0.05, compared with ADP control, ^Δ^ *P* < 0.05, compared with THR control; (D) *P < 0.05, compared with TRAP control.

**Figure 3:**
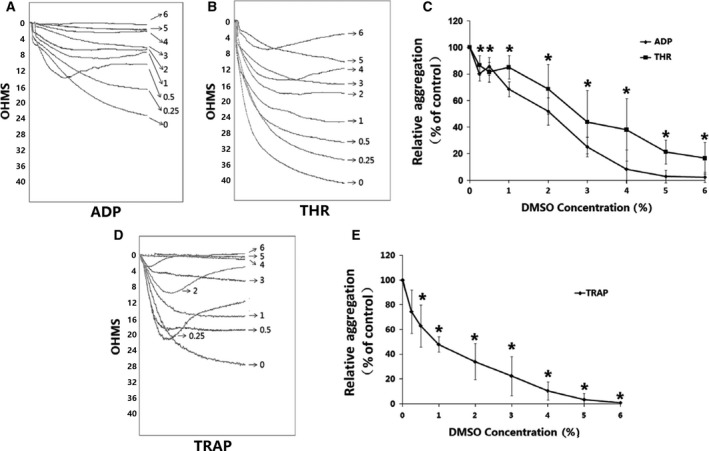
DMSO reduces aggregation rates of PLT in whole blood *in vitro*. DMSO concentrations were from 0.25% to 6%, and incubation time was 30 min. Controls were incubated without DMSO. Three aggregation stimuli ADP 10 U L^−1^, THR 1 U L^−1^ and TRAP 10 μm were used. (A, B, D) ADP, THR, TRAP were added to PLTs incubated with DMSO at different concentrations. Then samples were analyzed by impedance aggregometry. (C, E) Quantitative data from three experiments are shown (mean ± SD). *P < 0.05, compared with control.

The effects of DMSO (0.25–6%) on the PLT aggregation function of samples stimulated with ADP, THR, or TRAP were determined by light transmission and impedance aggregometry (Figs. 2 and 3). Inhibition of PLT aggregation by DMSO was evident. The results showed that DMSO reduced ADP, THR, and TRAP‐induced PLT aggregation starting at concentrations of 0.5%, 2%, and 0.25%, respectively. DMSO increased PLT aggregation inhibition rates in a dose‐dependent manner. When the concentration of DMSO was 6%, the inhibition rates of ADP, THR, and TRAP‐induced PLT aggregation by light transmission aggregometry were 91.70 ± 6.40%, 90.5 ± 2.12%, and 100%, and the inhibition rates by impedance aggregometry were 97.78 ± 3.85%, 83.45 ± 11.95%, and 98.72 ± 2.22%, respectively.

**Figure 4:**
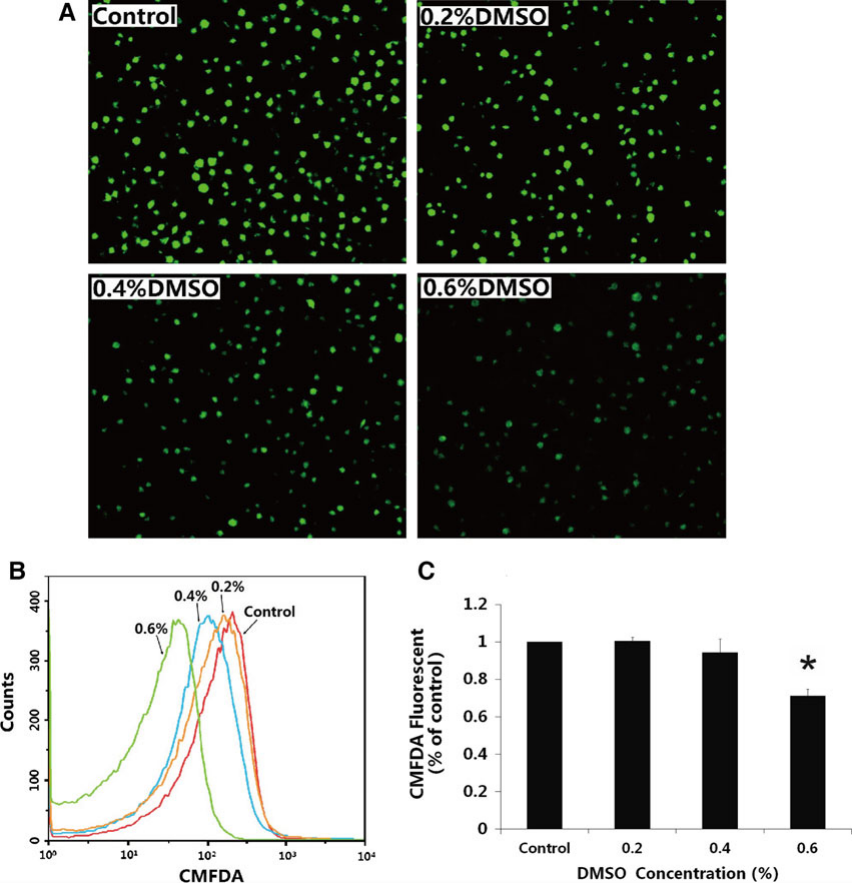
DMSO reduces intensities of cytosolic esterase‐induced fluorescence response in PLTs *in vitro*. DMSO concentrations were 0.2%, 0.4%, and 0.6%, incubation time were 30 min. Controls were incubated without DMSO. (A) CMFDA was added to PLTs incubated with DMSO at different concentrations. Then samples were incubated in the dark at 37 °C for 20 min and analyzed by laser confocal scanning microscope. (B) The samples were incubated in the dark at 37 °C for 20 min and analyzed by flow cytometry. (C) Quantitative data from three experiments are shown (mean ± SD). *P < 0.05, compared to control.

To determine how DMSO triggered the toxicity response on PLTs, effects of DMSO (0.2%, 0.4%, and 0.6%) were studied on the PLT fluorescence intensity response (Fig. 4). CMFDA fluorescent dye has been designed to freely pass through cell membranes into cells, where it is transformed into cell membrane‐impermeant reaction products. CMFDA dye is retained in living cells through several generations. It has been proved that the cytosolic esterase‐induced fluorescence intensity from CMFDA in PLTs is related to PLT function 27. As expected, the results showed that DMSO significantly down‐regulated PLT cytosolic esterase‐induced fluorescence intensity from CMFDA. CMFDA fluorescence intensity decreased 29% ± 0.04% with 0.6% DMSO (*P* < 0.05).

**Figure 5:**
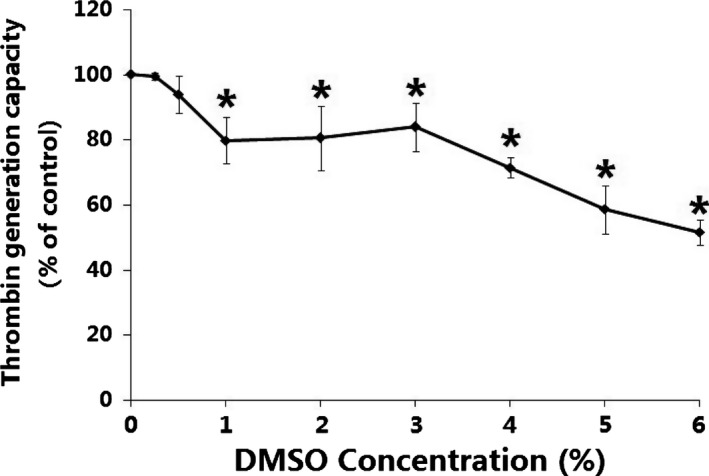
THR generation capacities in whole blood are reduced *in vitro* after DMSO. DMSO concentrations were from 0.25% to 6%, incubation time were 30 min. Controls were incubated without DMSO. Samples were quantified spectrophotometrically at 405 nm with a 96‐well plate reader. Quantitative data from three experiments are shown (mean ± SD). *P < 0.05, compared with control.

The effect of DMSO on THR generation is shown in Fig. 5. As the DMSO concentration was gradually raised from 0.25% to 6%, the THR generation concentration decreased. The results showed that the THR generation capacity was decreased after PLTs incubated with DMSO.

CD41a, also known the CD41/CD61 complex (GPIIb/IIIa), is a receptor for fibronectin, fibrinogen, von Willebrand factor, vitronectin, and thrombospondin and mediates PLT aggregation. The extent of PLT apoptosis was represented by the expression of PS. In our experiments, the levels of CD41a and apoptosis in PLTs did not have any significant differences between control group and DMSO incubation groups (data not shown).

### EAhy926 cells' proliferation was inhibited and apoptosis was increased after incubation with DMSO

**Figure 6:**
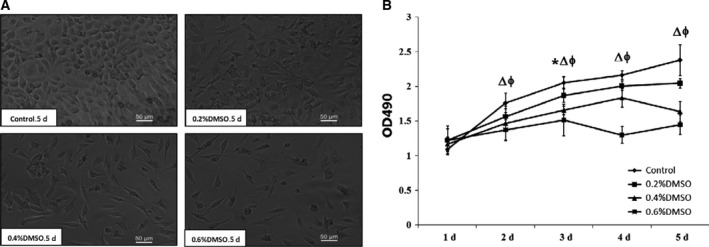
DMSO inhibits proliferation in EAhy926 cells. DMSO concentrations were 0.2%, 0.4%, and 0.6%, and controls were incubated without DMSO. DMSO was added to the cell culture medium, and then the cells were cultured for 5 days. (A) Samples were observed by optical microscope on the 5th day. (B) Quantitative data from four experiments are shown (mean ± SD). *P < 0.05, ^Δ^ *P* < 0.05, and ^ϕ^ *P* < 0.05, for 0.2%, 0.4%, and 0.6% DMSO, respectively, compared with control.

**Figure 7:**
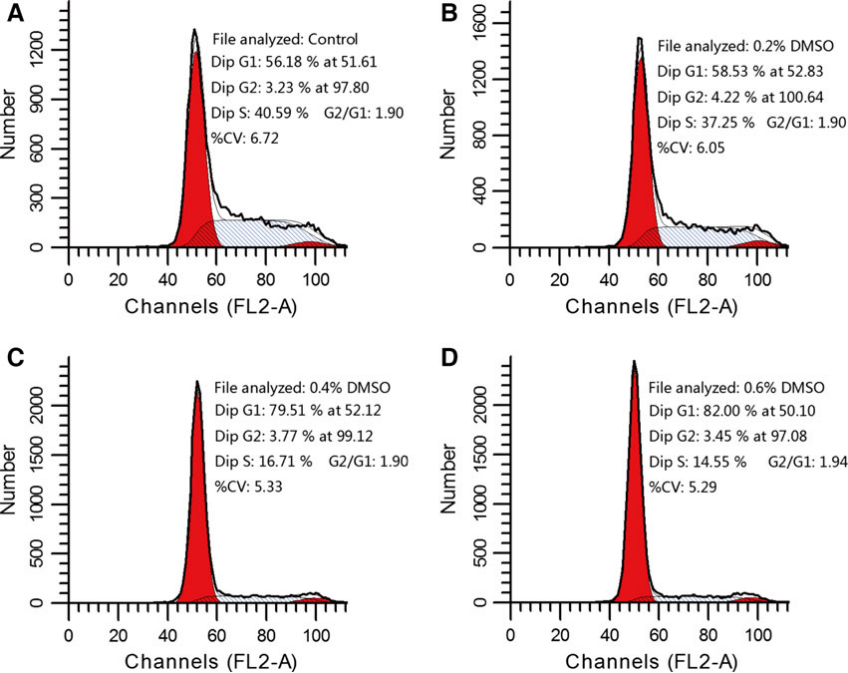
DMSO prevents growth in EAhy926 cells from G1 phase to S phase. DMSO concentrations were 0.2%, 0.4%, and 0.6%, and controls were incubated without DMSO. DMSO was added to the cell culture medium, and then the cells were cultured for 24 h. The G1 phase level of EAhy926 cells cocultured with DMSO (C, D) was significantly increased compared with control (A). Results are presented from one representative experiment. The same experiment was repeated at least three times.

**Figure 8:**
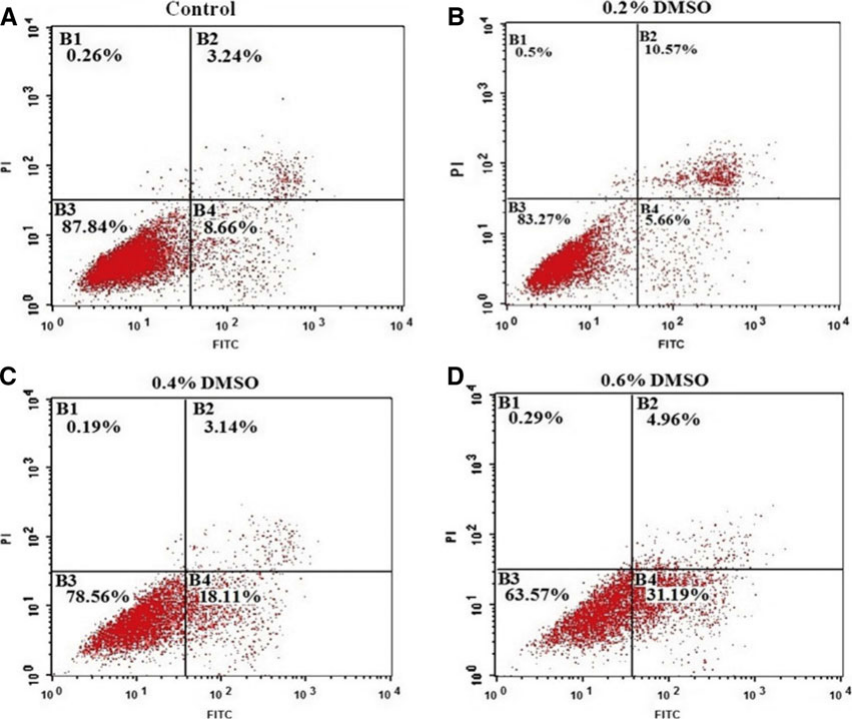
DMSO induces apoptosis in EAhy926 cells. DMSO concentrations were 0.2%, 0.4%, and 0.6%, and controls were incubated without DMSO. DMSO was added to the cell culture medium, and then the cells were cultured for 5 days.The total apoptosis (including early and late phase apoptosis) of EAhy926 cells cocultured with DMSO (B, C, D) was significantly increased compared with control (A). Results are presented from one representative experiment. The same experiment was repeated at least three times.

Figure 6 shows that, at the concentration range tested, DMSO had effects on the proliferation of EAhy926 cells. With the addition of DMSO 0.2%, 0.4%, and 0.6% to the culture medium for five consecutive days, the optical density (OD) at 490 nm of EAhy926 cells was 2.04 ± 0.07, 1.63 ± 0.14, and 1.44 ± 0.14, respectively, that of the control cells cultivated in the medium without DMSO (2.37 ± 0.23), which suggests that proliferation of EAhy926 cells could be decreased by the addition of DMSO. To clarify how DMSO inhibits the proliferation of EAhy926 cells, we studied the cycle of EAhy926 cells cultured for 24 h, and found that DMSO inhibited cell growth in the G1 phase, preventing progression to the S phase (Fig. 7). The present data show that the apoptosis of EAhy926 cells in cell culture media with DMSO stimulation (0.2%, 0.4% and 0.6%) was increased by almost 0.61 ± 0.21‐fold, 1.30 ± 0.55‐fold, and 2.03 ± 0.12‐fold, respectively, compared to the control (Fig. 8).

## Discussion

DMSO has been used for decades as a cryoprotective agent in biological studies 28, 29. In studies of PLT cryopreservation, researchers mainly focused on survival and recovery 3, 30. Other impacts of PLT transfusion from donor to recipient have not been explored. The FDA has not approved DMSO‐cryopreserved PLTs for clinical use. The total number of PLTs that were transfused were most likely greater in patients who received the liquid‐preserved PLTs than in the patients who received the previously cryopreserved PLTs. However, cryopreserved PLTs are significantly more effective than liquid‐preserved PLTs for controlling perioperative bleeding and amend the introduction accordingly with a more comprehensive review of other clinical studies 31, 32. Despite frequent use of DMSO, little is known about how this compound acts on the infusion response at the cellular levels of the hematologic system. Therefore, the aim of this work was to examine whether DMSO exhibits some toxic dose‐dependent effects on RBCs, PLTs, and vascular endothelial cells *in vitro*.

Many toxicological and clinical studies have been performed to determine the safety of DMSO. In our experiments, DMSO caused RBC hemolysis; this is consistent with a report of hemolysis in animals that received DMSO intravenously 9. There are mainly three mechanisms underlying the external substance‐induced hemolysis, oxidative hemolysis, immune hemolysis as well as nonimmune hemolysis 33. The main cause of oxidative hemolysis is that RBCs are extremely sensitive to oxygen‐derived free radicals, easily lead to functional damage and shortening the life. However, it has reported that DMSO has the characteristics of antioxidant effects, so RBCs’ hemolysis by oxidative damage cannot be caused by DMSO 34. Immune hemolysis refers that external substances after into the body can cause antibody‐mediated hemolysis through a variety of immune mechanisms 16. Most of the drugs injected into the body such as methyldopa and penicillin can cause immune hemolysis. Nonimmune hemolysis refers that the external substances by changing blood homeostasis caused RBCs aggregation and hemolysis, generally dose‐related. We have shown that DMSO‐induced hemolysis belongs to the dose‐related non‐immune hemolysis. The results in guinea pig RBCs showed that the external substances of non‐immune hemolysis could lead to RBCs’ morphological changes, shrinkage, and even fragmentation, suggesting that there may be a direct hemolytic risk of the external substances. A certain concentration of DMSO influenced the stability of RBCs by changing RBCs’ internal and external material imbalance, causing acute adverse reactions.

Platelet as one of the main components in the hematological system plays an irreplaceable role in the human coagulation function. The results showed that PLT aggregation *in vivo* was inhibited by DMSO in a dose‐dependent manner, we found a negative correlation between PLT aggregation and DMSO concentration. The expressions of membrane GPIIb/IIIa (CD41a) and membrane PS in PLTs were not significantly influenced after DMSO treatment. The secretion of platelet microparticles (PMPs) which played an important role in PLT aggregation did not increase. However, the capacity of endogenous THR generation was found to be reduced, we hypothesized that DMSO may be involved in a binding‐site competion with THR, which helps to reduce PLT aggregation.

It was reported previously that DMSO induced significant apoptotic changes in PC12 cells, whereas cytotoxic effects were observed with DMSO concentrations ≥4% 35. For example, a low concentration of DMSO prevented gastric cancer cell progression from G0/G1 to S phase, thus inhibiting the cell growth 36. EAhy926 cells are hybridoma cell lines from umbilical vascular endothelial cells and lung cancer cells, and have the properties of vascular endothelial cells 37. A low concentration of DMSO inhibited the growth of EAhy926 cells, prevented progression from G1 phase to S phase, and elevated apoptosis rates. Vascular endothelial cells are the first protective barrier when DMSO is infused into the body. Therefore, with DMSO penetrating biological membranes rapidly, vascular endothelial cells are extremely vulnerable.

In patients with coronary artery disease, infusion of autologous stem cells cryopreserved with DMSO can cause respiratory arrest, but they can be reinfused into the body without the appearance of further complications after DMSO is washed out 31. For hematologic diseases and other medical treatments in which blood transfusion is needed, frozen blood products containing DMSO are the ‘second line of attack’ for patients. Because patients given cryopreserved blood products are at high risk for graft infusion‐related reactions, maximum effort should be made to prevent and interfere with these complications. We recommend that blood cell products be washed prior to infusion to deplete DMSO.

In summary, DMSO at low concentrations showed toxic properties in the blood cells, inducing RBCs hemolysis, reducing PLT activities, decreasing PLT aggregation levels, inhibiting the growth of EAhy926 cells, preventing cell progression from G1 phase to S phase, and elevating apoptosis rates. In this study, we cannot rule out the possibility that DMSO plays an important role in hematologic damage via its toxic properties, but more mechanistic investigations are necessary to better understand how this compound influences the metabolic system.

## Author contributions

XYY, JXW, and YH conceived and designed the experiments, analyzed the data, and wrote the paper. XYY, JXW, MXL, and HC performed the experiments. XYY, JXW, YH, HLZ, and QL contributed reagents/materials/analysis tools.
